# Astaxanthin attenuates cognitive deficits in Alzheimer’s disease models by reducing oxidative stress via the SIRT1/PGC-1α signaling pathway

**DOI:** 10.1186/s13578-023-01129-w

**Published:** 2023-09-14

**Authors:** Ning Liu, Xiaohong Lyu, Xianglin Zhang, Fan Zhang, Yiming Chen, Gang Li

**Affiliations:** 1 Department of Radiology, The First Affiliated Hospital of Jinzhou Medical University, Jinzhou, 121001 China; 2 Department of Neurology, The First Affiliated Hospital of Jinzhou Medical University, Jinzhou, 121001 China

**Keywords:** Astaxanthin, Aβ25–35, Oxidative stress, Alzheimer’s disease, Cognitive, Cellular, Senescence, Apoptosis

## Abstract

**Objective:**

Oxidative stress plays a pivotal role in neurodegenerative diseases. Astaxanthin (AST) can play a neuroprotective role owing to its long-chain conjugated unsaturated double bond, which imparts potent antioxidant, anti-neuroinflammatory, and anti-apoptotic properties. However, the biological mechanisms underlying these effects remain unknown. Therefore, this study aimed to investigate and validate the protective effect of AST on neuronal senescence and apoptosis caused by oxidative stress induced by Aβ25–35 peptide, with the goal of preventing the onset of cognitive dysfunction.

**Methods:**

Alzheimer's disease models comprising ICR mice and PC12 cells were established using Aβ25–35. The Morris water maze test was used to assess mouse behavior. Nissl staining revealed morphological changes in the mouse hippocampal neurons. To elucidate the mechanism of action of AST, ICR mice and PC12 cells were treated with the silent information regulator 1 (SIRT1) inhibitor nicotinamide (NAM). Additionally, immunofluorescence, western blotting, and reverse transcription polymerase chain reaction were used to evaluate changes in the expression of Bcl-2 and Bax in the mouse hippocampus, and SIRT1/PGC-1α signaling pathway proteins were detected. Moreover, the oxidative stress markers in ICR mice and PC12 cells were evaluated. Further, CCK-8 assays, Annexin V/PI double staining, and β-galactosidase activity assays were performed in PC12 cells to evaluate the anti-senescence and apoptotic effects of AST.

**Results:**

In vivo experiments showed that Aβ25–35 impaired cognitive function, promoted morphological changes in hippocampal neurons, decreased Bcl-2 expression, increased Bax expression, decreased superoxide dismutase and GSH-px levels, and increased reactive oxygen species and malondialdehyde levels. Conversely, AST alleviated the impact of Aβ25–35 in mice, with reversed outcomes. NAM administration reduced SIRT1 and PGC-1α expression in the hippocampus. This decrease was accompanied by cognitive dysfunction and hippocampal neuron atrophy, which were also evident in the mice. Additionally, in vitro experiments showed that Aβ25–35 could promote oxidative stress and induce the senescence and apoptosis of PC12 cells. Nonetheless, AST treatment counteracted this effect by inhibiting oxidative stress and altering the state of PC12 cells. Notably, the Aβ + NAM group exhibited the most significant rates of senescence and apoptosis in PC12 cells following NAM treatment.

**Conclusion:**

AST can improve cellular senescence and apoptosis mediated by oxidative stress via the SIRT1/PGC-1α signaling pathway and plays a vital role in inhibiting neuronal senescence and apoptosis and enhancing cognitive ability.

## Introduction

The biological mechanisms and consequences of aging have captured the attention of numerous scholars, especially considering the rise in life expectancy. Aging is characterized by a decline in the ability to adapt to stress, an increase in reactive oxygen species (ROS), a reduction in cellular bioenergy, and an increased risk of age-related diseases, such as Alzheimer's disease (AD), which impairs cognitive function [1]. Moreover, oxidative stress accelerates the processes of aging and AD [2–5]. Oxidative stress plays a pivotal role in numerous chronic diseases, including neurodegenerative diseases where amyloid beta (Aβ) plaques develop in the brain tissues [6–8]. Accumulating evidence suggests that Aβ inhibits ATP synthesis and generates excess ROS. Additionally, Aβ exposure induces increased ROS production in neurons, resulting in diminished cognitive function owing to neuronal apoptosis [9, 10].

Anti-apoptotic proteins, including B-cell lymphoma-2 (Bcl-2), are triggered by apoptosis in acute and chronic degenerative diseases, leading to the downregulation or modification of pro-apoptotic proteins such as Bcl-2-associated X (Bax). Throughout all stages of neurodevelopment, both Bax and Bcl-2 are expressed [11–15]. According to previous studies, these proteins regulate cell death in neurons, granuloma cells, and Purkinje cells. Additionally, silent information regulator 1 (SIRT1) is another crucial player in combating the detrimental effects of increased ROS. SIRT1 can regulate intracellular ROS levels and prevents cell degeneration caused by increased ROS [16–19]. Moreover, peroxisome proliferator-activated receptor γ coactivator-1α (PGC-1α) exerts influence on intracellular mitochondrial activity and the mobilization of cell-reinforcing catalysts, thereby enhancing cellular resilience against ROS [16, 20, 21]. The SIRT1/PGC-1α signaling pathway is essential for neuronal development, repair of damage, and neuroprotection during brain aging [16, 17] owing to its potent antioxidant attributes [16, 17].

Recent research has extensively focused on PC12 cells as models for exploring neuronal physiological and biochemical components facets [22, 23]. PC12 cells are derived from pheochromocytomas of the adrenal medulla in rodents and can differentiate into “intelligent” neurons [24]. Astaxanthin (AST) has emerged as a notable subject of study [25] owing to its potent cell-reinforcing properties. AST affects ROS levels, which maintains and enhances mitochondrial activity in neurons, reduces DNA damage and aggravation, and prevents oxidative stress-induced cell damage and death [26, 27]. Moreover, a few exceptional compounds, including AST, can cross the blood–brain barrier and protect neurons from damage and apoptosis [28, 29].

To date, the protective effect of AST on cellular senescence and apoptosis via the SIRT1/PGC-1α signaling pathway has not been reported. The subjects of this study were ICR mice and PC12 cells, and these were induced by Aβ25–35. We aimed to investigate the protective effect of AST against hippocampal neuronal apoptosis and PC12 cell senescence and apoptosis, thereby suppressing cognitive dysfunction. This study provides an important theoretical basis for using AST as a therapeutic candidate for the treatment of neurodegenerative diseases.

## Materials and methods

### Animals

Eighty male ICR mice (6 weeks old, weighing 20–25 g) were provided by Liaoning Changsheng Co. Ltd (SCXK-2020-001). These mice were fed standard laboratory chow and water (55 ± 5% humidity, 23 ± 2 °C, and 12-h light/dark cycle). Additionally, the mice were subjected to humane laboratory procedures according to the guidelines for the Use and Care of Laboratory Animals. Steps were taken to alleviate the suffering of the mice during the experiments and adhere to the ethical standards and regulations of Jinzhou Medical University. This study was approved by the Animal Ethics Committee, Jinzhou Medical University, China.

### Animal and AD models

**Fig. 1:**
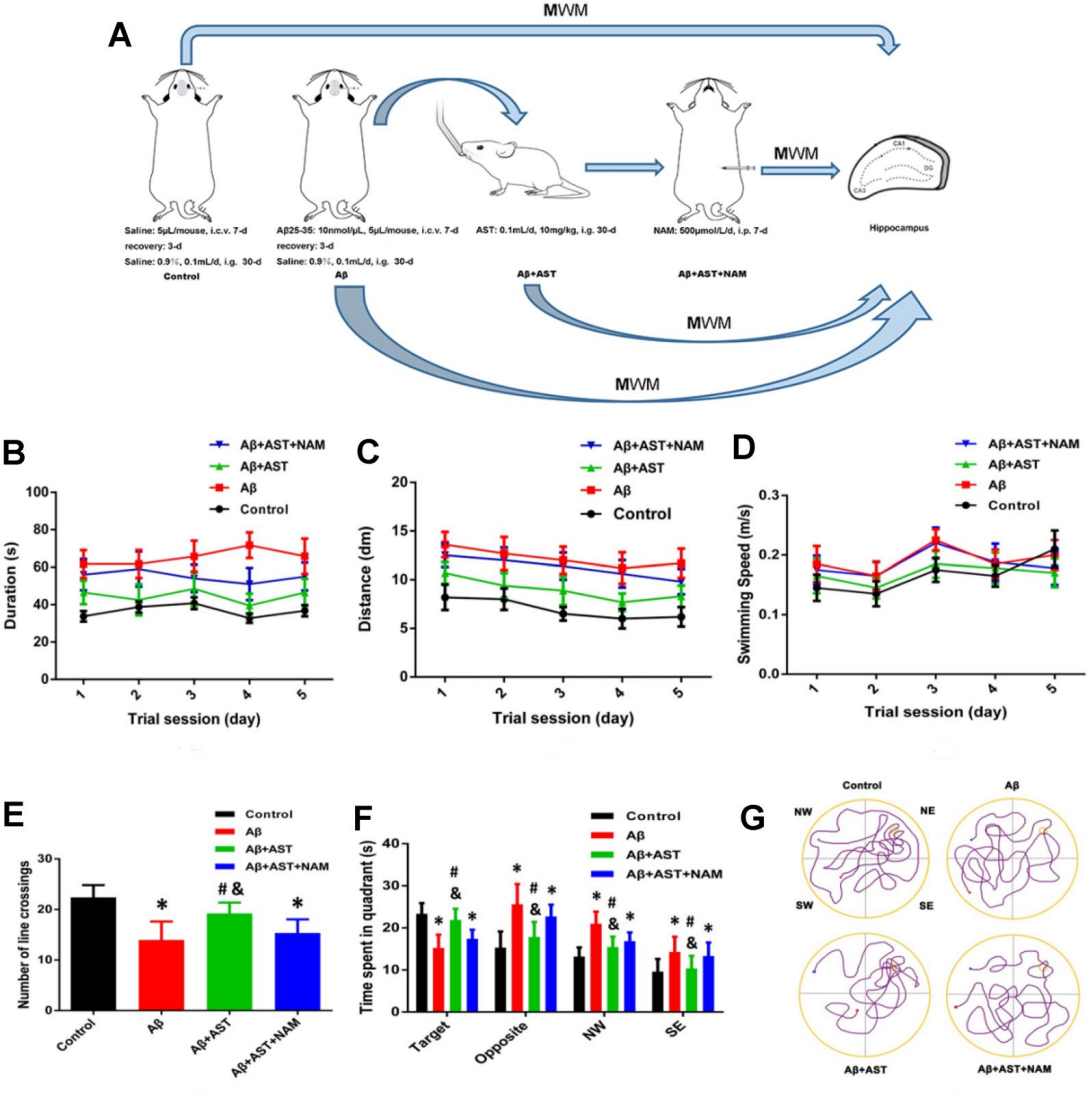
The mice in the control, Aβ, Aβ + AST, and Aβ + AST + NAM groups were treated with the corresponding reagents (n = 20/group). After successful modeling, the Morris water maze test was performed on each group of mice. **A** Schematic diagram of the experiment process. **B** Duration of each group of mice. **C** Swimming distance of each group of mice. **D** Swimming speed of each group of mice. **E** The number of times that mice in 4 groups crossed the platform in the probe experiment. *P < 0.05, Control group vs. Aβ group and Aβ + AST + NAM group. #P < 0.05, Aβ + AST group vs. Aβ + AST + NAM group. &*P* < 0.05, Aβ + AST group vs. Aβ group. **F** The time in quadrants of 4 groups of mice. *P < 0.05, Control group vs. Aβ group and Aβ + AST + NAM group. #P < 0.05, Aβ + AST group vs. Aβ + AST + NAM group. &*P* < 0.05, Aβ + AST group vs. Aβ group. **G**: The escape latency of the four groups of mice in the positioning navigation experiment

The experimental mice were randomized and separated into four groups: the control group, Aβ group, Aβ + AST group, and Aβ + AST + nicotinamide (NAM) group (n = 20 per group). Animal randomization was conducted using http://randomisation.com/. Mice in the control group were treated with a right lateral ventricle saline injection (5 μL/mouse). The Aβ group received right lateral ventricle injections of Aβ25–35 (10 nmol/μL, 5 μL/mouse, Sigma, USA), and the time of administration was 9:00 am on the 2nd, 4th, 6th, 8th, 10th, 12th, and 14th days, totaling seven injections [30]. The Aβ + AST and Aβ + AST + NAM groups received AST treatment by oral gavage (0.1 mL/d, 10 mg/kg, Sigma, USA) for 30 consecutive days following a 3-day recovery period. Meanwhile, the mice of the control group and the Aβ group received sterile saline (0.9%, 0.1 mL/d) by oral gavage for 30 consecutive days following a 3-day recovery period. Furthermore, the Aβ + AST + NAM group received intraperitoneal injections of NAM (500 μM/d, Sigma, USA) for 7 consecutive days after AST treatment. The schematic representation of model creation is illustrated in Fig. 1A.

### Behavioral tests

The Morris water maze test (MWM; Any-maze, Stoelting, USA) was employed to evaluate spatial memory and learning abilities during the model preparation [31]. The mice were placed in a swimming pool for 2 min on the first day (away from the platform) to acclimatize them to the maze environment. The experiment was conducted four times daily for 5 consecutive days.

During each trial, the mice were given 120 s to locate the submerged platform. If successful, they were allowed to remain on the platform for 20 s. However, if a mouse failed to find the platform within 120 s, it was gently guided to the platform and left there for 20 s. The time taken for each mouse to find the platform during each trial, known as the "escape latency time," was recorded, along with its swimming path and speed.

On the 6th day, the probe experiment commenced, involving the removal of the platform. Before concluding the training, the mice were given 120 s to swim freely. In this computer-based probing experiment, indicators of spatial memory ability were measured, including swimming speed, the number of passes through the platform quadrant, and the time taken to locate the platform.

### Tissue preparation

Following preliminary tests, mice from each group were euthanized under anesthesia with avertin (1.25%, 500 μL intraperitoneally). Subsequently, their brains were rapidly removed and halved. Next, hippocampal tissues from single slice of the brains were implanted in paraffin and divided into 5-μm thick sections for immunofluorescence. Additional hippocampal fragments were prepared for western blot analysis, immediately frozen in liquid nitrogen, and stored at − 80 °C.

### Cell preparation

PC12 cells with fewer than seven passages were utilized in the experiments. Counting and digesting PC12 cells in the logarithmic growth stage produced a cell suspension with a concentration of 6 × 10^4^ cells/mL. Each well of the 96-well cell culture plate was loaded with 100 μL of the cell suspension, which was then cultivated for 24-h at 37 °C in an incubator with 5% CO_2_. After the cells had completely adhered to the culture medium, the experimental group was added to the culture medium and diluted with 100 μL of Aβ25–35 at concentrations of 1 μM, 5 μM, 10 μM, 20 μM, 40 μM, 80 μM, and 160 μM in each well at 37 °C. The control group was established by adding the same volume of DMEM medium after 48-h of culture in the 5% CO_2_ incubator [32].

CCK-8 (KGA 317, Jiangsu KGI Biotechnology Co., LTD, Jiangsu, China) was used to stain the 96-well plates. After adding 10 μL of CCK-8 to each well, the culture was incubated for another 2-h. The plate was then shaken and gently mixed for 10 min. Subsequently, the optical density (OD) value was measured, and the cell inhibition rate was determined using at least three samples at a time using a microplate reader, the meter set at 450 nm.

The experimental dose concentration of Aβ25–35 was set at 20 μM based on the CCK-8 detection data. Experimental groups: control group (DMEM medium); Aβ group (Aβ 20 μM, 48-h); AST group (AST 10 μM, 48-h); Aβ + AST group (Aβ 20 μM + AST 10 μM, 48-h); Aβ + AST + NAM group (Aβ 20 μM + AST 10 μM + NAM 5 μM, 48-h); Aβ + NAM group (Aβ 20 μM + NAM 5 μM, 48-h). The viability of PC12 cells in each group was detected using the CCK-8 assay.

### Immunofluorescence staining analysis

Paraffin slices were dewaxed and hydrated to obtain hippocampal tissues. The sections were permeabilized for 10 min at 25 ℃ using 0.5% TritonX-100. The slices were then washed with PBS for 3 times and incubated with 10% goat serum for 30 min at 25 ℃. Subsequently, the slices were incubated with diluted primary antibodies: anti-Bcl-2 (1:500, #12,789, PTG, Rosemont, USA), anti-Bax (1:500, #50,599, PTG, Rosemont, USA), anti-SIRT1 (1:500, #13,161, PTG, Rosemont, USA), and anti-PGC-1α (1:500, #20658, PTG, Rosemont, USA) at 4 °C overnight in a refrigerator. On the following day, washing with PBS for 5 times, the slices were subjected to incubation with a secondary antibody (goat anti-rabbit) and incubated for 1-h in the dark at 25 ℃. The slices were then placed in PBS and washed thrice before incubation with 0.0033% H_2_O_2_ and 0.025% 4',6-diamino-2-phenylindole (DAPI) in PBS for 10 min. Subsequently, the slices were flushed with PBS to remove excess DAPI. The slices were mounted with Vectashield HardSet Antifade Mounting Medium (H-1400, Vector Laboratories, Burlingame, CA, USA) and visualized using a fluorescence microscope (DMC6200, Leica, Germany) [31]. The average fluorescence intensities were measured and evaluated using Image J software (National Institutes of Health, Bethesda, MD, USA).

### Western blot analysis

Protein levels of Bcl-2, Bax, SIRT1, and PGC-1α in the hippocampal samples were evaluated using western blotting according to standard protocols. Briefly, protein samples were transferred onto PVDF membranes (Millipore, Billerica, MA, USA). Subsequently, the membranes were incubated with primary antibodies: anti-Bcl-2 (1:500, #12789, PTG), anti-Bax (1:500, #50599, PTG), anti-SIRT1 (1:500, #13161, PTG), anti-PGC-1α (1:500, #20658, PTG), and appropriate secondary antibodies. GAPDH (1:1000, #10494, PTG) was used as an internal loading control. Subsequently, the membranes were observed using an enhanced chemiluminescence solution [31]. Finally, the band intensities were analyzed using Image J software.

### Real-time PCR analysis

Primer sequences were obtained from Nanjing Kingsrui Biotechnology Co., LTD. Jiangsu, China. Bcl-2: 5′-GAGCCTGTGAGAGACGTGG-3′(forward) and 5′-CGAGTCTGTGTATAGCAATCCCA -3′ (reverse); Bax: 5′-AGACAGGGGCCTTTTTGCTAC-3′(forward) and 5′-AATTCGCCGGAGACACTCG-3′ (reverse); SIRT1: 5′-TGATTGGCACCGATCCTCG-3′(forward) and5′-CCACAGCGTCATATCATCCAG-3′ (reverse); PGC-1α: 5′-TATGGAGTGACATAGAGTGTGCT-3′(forward) and 5′-GTCGCTACACCACTTCA. ATCC-3′(reverse); GAPDH:5′-ACTTTGGCATCGTGGAAGGG-3′(forward) and 5′-ACTTGGCAGGTTTC. TCCAGG-3′(reverse). The amplification conditions were as following: denaturation at 95 ℃ for 30 s, amplification at 95 ℃ for 5 s, followed by 60 ℃ for 20 s, totaling of 40 cycles. Subsequently, the reaction was subjected to a melt curve analysis by heating at 95 ℃ for 10 s, then at 65 ℃ for 10 s, and finally cooling at 40 ℃ for 30 s. The reaction was performed using an Eppendorf Master Cycler Realplex real-time fluorescence quantitative PCR instrument, and the results represent the relative expression levels of the target mRNA standardized by the internal reference GAPDH. The experiment was repeated three times, and the mean value was calculated. The amplification reaction system consisted of the following components: SYBR Premix Ex Taq II (10 μL), PCR forward primer (0.4 μL), PCR reverse primer (0.4 μL), cDNA (2.0 μL), and DEPC (7.2 μL), totalling 20 μL [31].

### Nissl staining analysis

Hippocampus of mice was removed and fixed in 4% paraformaldehyde over night, dehydrated with 30% sucrose in 4% paraformaldehyde, and then cut into 5-μm slices on a vibratome (VT1200S, Leica, Wetzlar, Germany). The sections were coated with gelatin and placed into chloroform:alcohol (1:1) overnight. The following day, the sections were dehydrated in graded ethanols (100%, 95%). The slices were stained with 0.1% cresyl violet acetate for 10 min, quickly rinsed in distilled water, and differentiated with 95% ethyl alcohol for 30 min. Sections then were dehydrated in graded ethanols and coverslipped with PermountTM. The slices were visualized and scanned using a slide scanner (Slide Scan System SQS1000, Teksqray, Guangzhou, China).

### Oxidative stress index analyses

#### Oxidative stress indexes in mice hippocampus

After taking hippocampal structures from each group, they were weighed and rinsed three times with 1:5 physiological saline. Protein lysis solution (RIPA) and PMSF were added proportionally, sonicated (30 s), and then lysed overnight at 4 ℃. The next day, they were centrifuged at low temperature at 12000 g for 20 min at 4 ℃. The supernatant was taken, and each tube was 200 μL sub packaging, 2 µl of each liquid were taken for BCA method to determine protein concentration, and a 10 µl protein system was established. The protein system contained 40 µg of protein, and 5 µl of protein were added 5 × loading buffer is 2 µl in total, which needs to be supplemented with PBS. After mixing, the protein denatures at 100 ℃ and is then placed in an EP tube −70 ℃ for storage. ROS content was evaluated using a DCFH-DA probe with an excitation wavelength of 488 nm and an emission wavelength of 525 nm (KGAF018, Jiangsu KGI Biotechnology Co., LTD, Jiangsu, China). Superoxide dismutase (SOD) content was determined using the four-mile nitrogen blue technique (NBT) and a UV spectrophotometer, with an absorption peak at 560 nm (A001-3–1, Jiangsu KGI Biotechnology Co., LTD). The thiobarbituric acid (TBA) method was used to measure malondialdehyde (MDA), and the amount was computed using an ultraviolet spectrophotometer following colorimetry with an absorption peak at 535 nm (KGT003, Jiangsu KGI Biotechnology Co., LTD). The glutathione peroxidase (GSH-Px) concentration was determined using an ultraviolet spectrophotometer following the colorimetric method with an absorption peak at 423 nm (KGT006, Jiangsu KGI Biotechnology Co., LTD). GSH-px levels were assessed using the colorimetric method (DTNB) [31].

#### Oxidative stress indexes in PC12 cells

In PC12 cells, using a microplate tester, intracellular ROS were detected using a DCFH-DA fluorescent probe at an excitation wavelength of 488 nm and an emission wavelength of 525 nm, and the ROS status of the cells was determined [32]. The preceding cell treatment procedures were carried out in accordance with the kit instructions to determine the SOD, GSH-Px, and MDA content in PC12 cells [32]. The amount of SOD was measured at λ = 540 nm using a microplate tester and the NBT technique. The OD value was then calculated to determine the SOD amount. Using a microplate tester and the DTNB technique, the amount of GSH-Px was detected at λ = 423 nm. Additionally, the OD value was calculated to determine the amount of GSH-Px present in the cells. MDA amount was measured at λ = 535 nm using a microplate tester and the TBA technique. Subsequently, the OD values were calculated to determine MDA amount.

#### Annexin V/PI double staining analysis

Digested PC12 cells in the logarithmic developmental stage were cultured in 6-well plates. As per the grouping of PC12 cells, the appropriate drug-containing medium dose was supplied 1 day after the cells adhered fully to the wall. In the control group, the same amount of DMEM was added, and the cells were digested and collected using 0.25% trypsin after 48-h of treatment (without EDTA). PC12 cells were centrifuged three times at 1000 × g for 5 min to harvest approximately 5 × 10^5^ cells. The cells were then suspended in 500 μL of binding buffer. To assess apoptosis, 5 μL of Annexin V-APC was added and mixed with the PC12 cells. The cells were incubated for 5–15 min at 25 ℃ and protected from light. Subsequently, signs of apoptosis were observed in each group of PC12 cells [33].

#### β-Galactosidase staining analysis

At pH 4–5, many cells produce β-galactosidase in lysosomes; however, at pH 6, β-galactosidase is only seen in senescent cells. Therefore, we stained PC12 cells in each group with a senescence-related β-Gal kit (KGPAG001, Jiangsu KGI Biotechnology Co., LTD, Jiangsu, China) to ascertain the statuses of the cells [34]. For the staining procedure, prepared cell climbing slides and lie them in the 24-well plates for backup, each well was inoculated with 500 μL of 4 × 10^4^ cells/mL, make the cells were put on slides. After the appropriate treatments and dosing, the cells were taken out of the incubator and prepared for staining. The β-galactosidase staining process involved applying 1 mL of fixative to each well, followed by a 15 min fixation at 25 ℃. The cell culture medium was then removed, and the fixed cells were washed thrice with PBS for 3 min each. After removing the PBS solution, 1 mL of staining solution (10 μL of solution A/10 μL of solution B/930 μL of solution C/50 μL of X-Gal solution) was added to each well, followed by incubation overnight in a temperature-controlled box at 37 ℃. On the following day, the cells were observed under an ordinary light microscope. Finally, the cell slides were removed and securely sealed with tablet sealants, and detailed images were meticulously captured under a microscope.

### Statistical analysis

All statistical analyses of variance were performed using GraphPad Prism 9.0 (GraphPad Software Inc., San Diego, CA, USA). All values are expressed as mean ± SD. Two-tailed independent *t*-tests were used to compare the two groups. Statistical significance was set at *P* < 0.05.

## Results

### AST improved learning, memory, and cognitive function in AD model

Results from the Morris water maze test revealed that mice in the Aβ group exhibited spatial learning and memory difficulties, taking longer to locate the submerged platform in the water. However, after AST treatment, the Aβ + AST group showed a significant improvement in learning and spatial memory capabilities. Conversely, the Aβ + AST + NAM group displayed a learning capacity similar to the Aβ group.

During the probe trial, the Aβ group spent less time in the platform quadrant and exhibited fewer track crossings than the control and Aβ + AST groups, indicating potential memory impairment in the Aβ group mice. In contrast, the Aβ + AST group exhibited a noteworthy decrease in the time required to reach the platform, along with an increase in the duration spent within the platform quadrant. Moreover, their performance showed a greater resemblance to the control group, with the experimental values closely corresponding. However, the results in the Aβ + AST + NAM group were comparable with those observed in the Aβ group. Therefore, the MWM analysis showed that AST can play an alleviating role in cognitive dysfunction and that AST may ameliorate cognitive dysfunction induced by oxidative stress through the SIRT1/PGC-1α signaling pathway. This conclusion was further verified by follow-up experiments (Fig. 1B–G).

### AST inhibited the apoptosis of hippocampus neurons in AD model

**Fig. 2:**
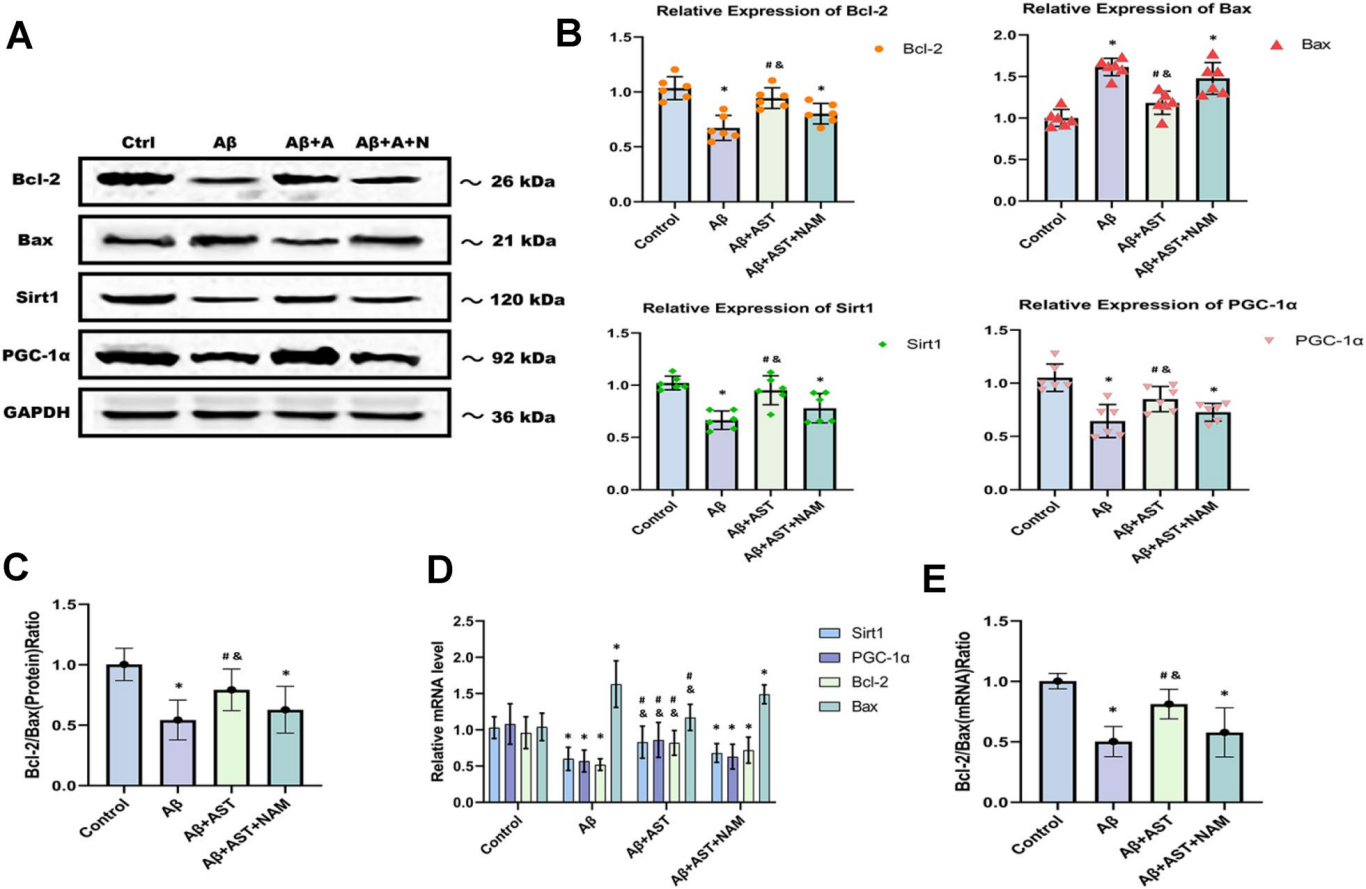
Bcl-2, Sirt1, and PGC-1α protein levels of expression were up-regulated by astaxanthin. Bax protein levels of expression were down-regulated by astaxanthin and up-regulated by Aβ25-35. **A** The expression bands of Bcl-2, Bax, SIRT1, and PGC-1α. **B** Analysis of expression changes of proteins. **C** The Bcl-2/Bax of proteins. **D** The level of mRNA changes of proteins. **E** The Bcl-2/Bax of mRNA.*P < 0.05, Control group vs. Aβ group and Aβ + AST + NAM group. #P < 0.05, Aβ + AST group vs. Aβ + AST + NAM group. &*P* < 0.05, Aβ + AST group vs. Aβ group. (n = 6/group)

When comparing protein levels of the Aβ group to Aβ + AST group using western blotting analysis, we found the protein level of Bax was elevated in the Aβ group, whereas the expression levels of Bcl-2, SIRT1, and PGC-1α were lower in the Aβ group (*P* < 0.05 for all variables). These findings suggest that AST increases the expression of Bcl-2, SIRT1, and PGC-1α in the hippocampus of mice while inhibiting the expression of Bax. The Aβ group had the lowest Bcl-2/Bax (protein) ratio compared with the control and Aβ + AST groups. Additionally, the Aβ + AST + NAM group had a significantly lower Bcl-2/Bax (protein) ratio than the Aβ + AST group (*P* < 0.05 for all variables) (Fig. 2A–C).

Using qRT-PCR, we identified the expression of *Bcl-2, SIRT1, PGC-1α*, and *Bax* mRNA in the mouse hippocampus samples. The expression trends were consistent with the western blot findings: the *Bcl-2/Bax* (mRNA) ratio in the Aβ group was lower than that in the control and Aβ + AST groups, whereas the ratio in the Aβ + AST group was lower than that in the Aβ + AST + NAM group (*P* < 0.05 for all variables) (Fig. 2D–E).

**Fig. 3:**
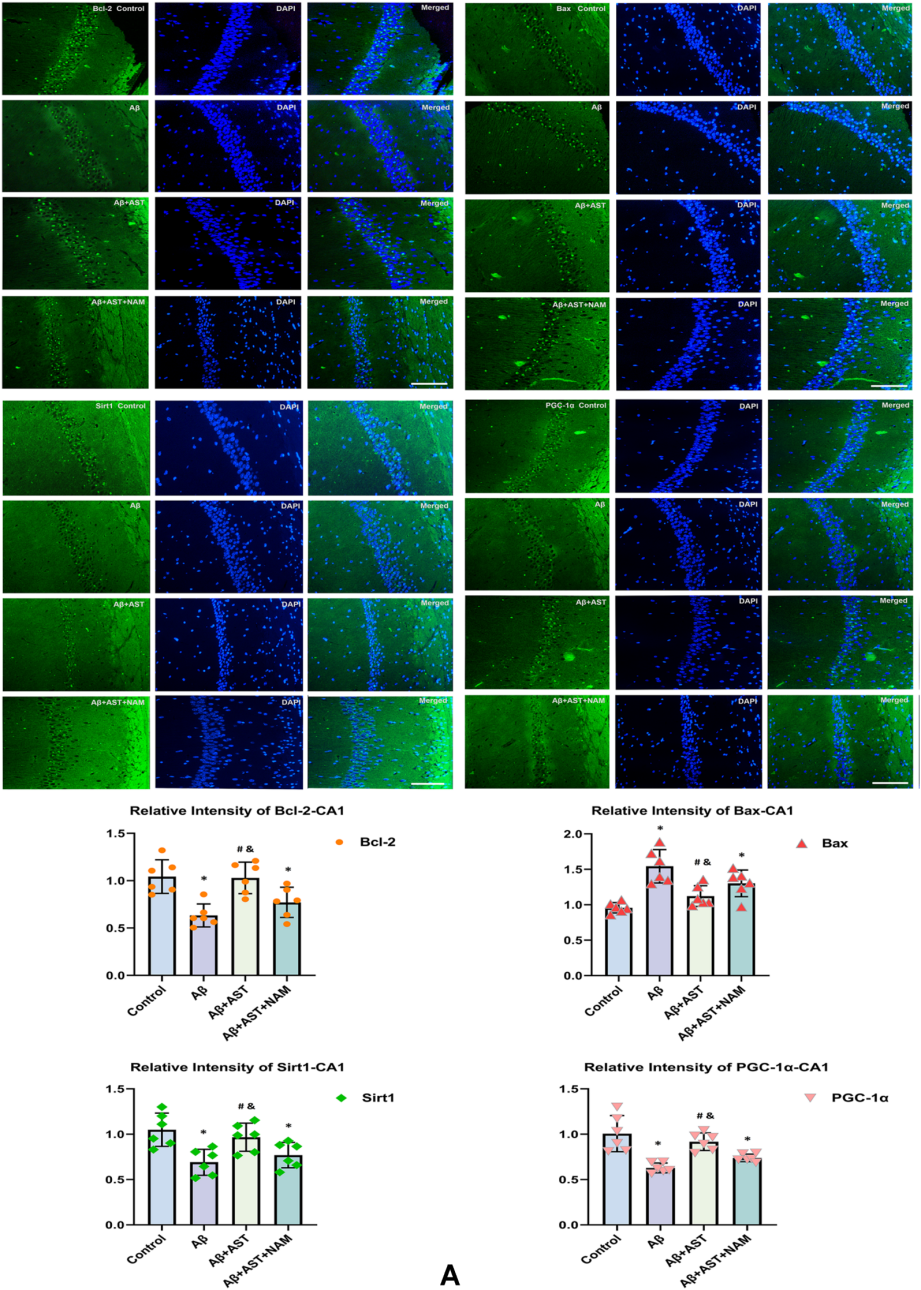

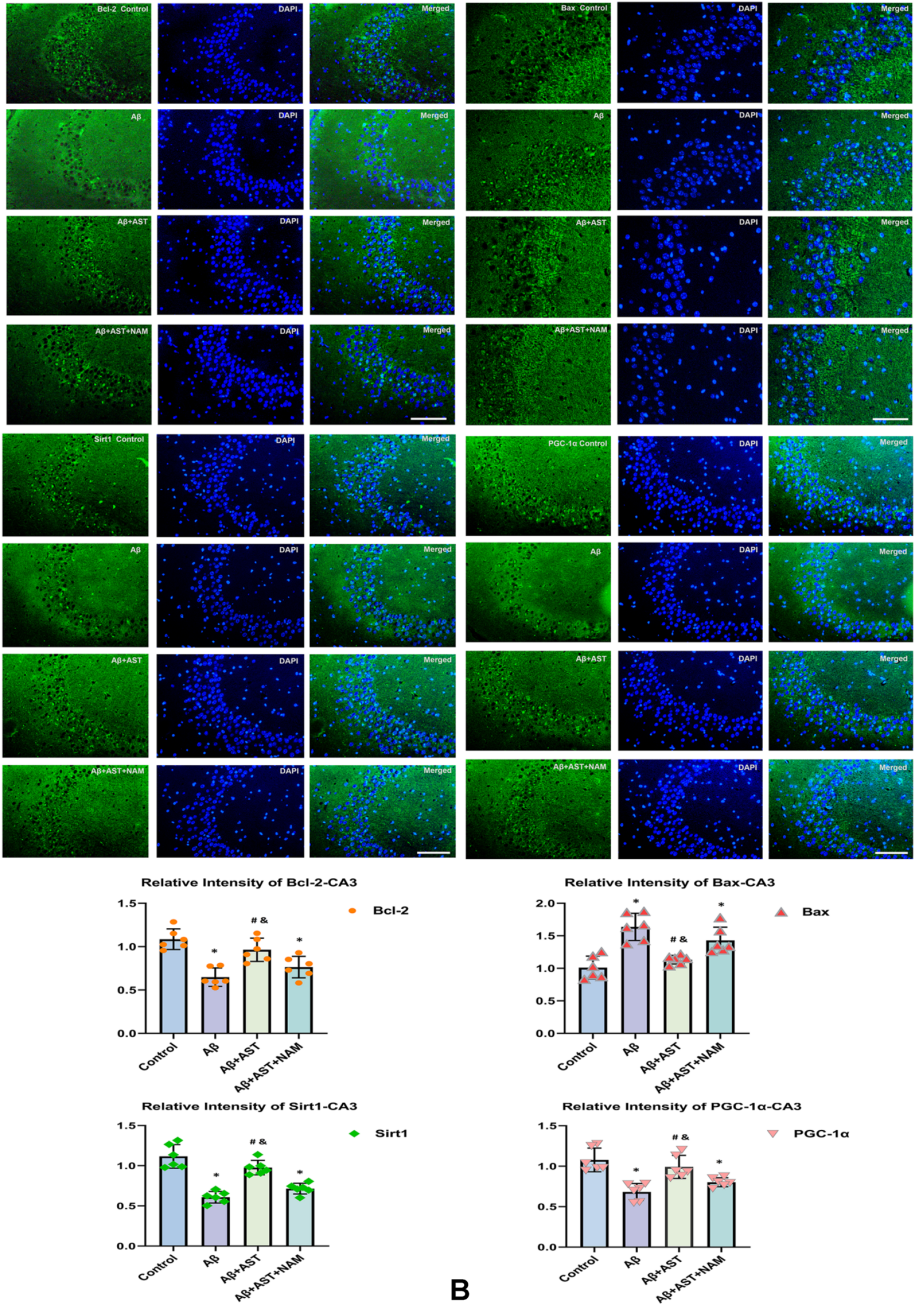

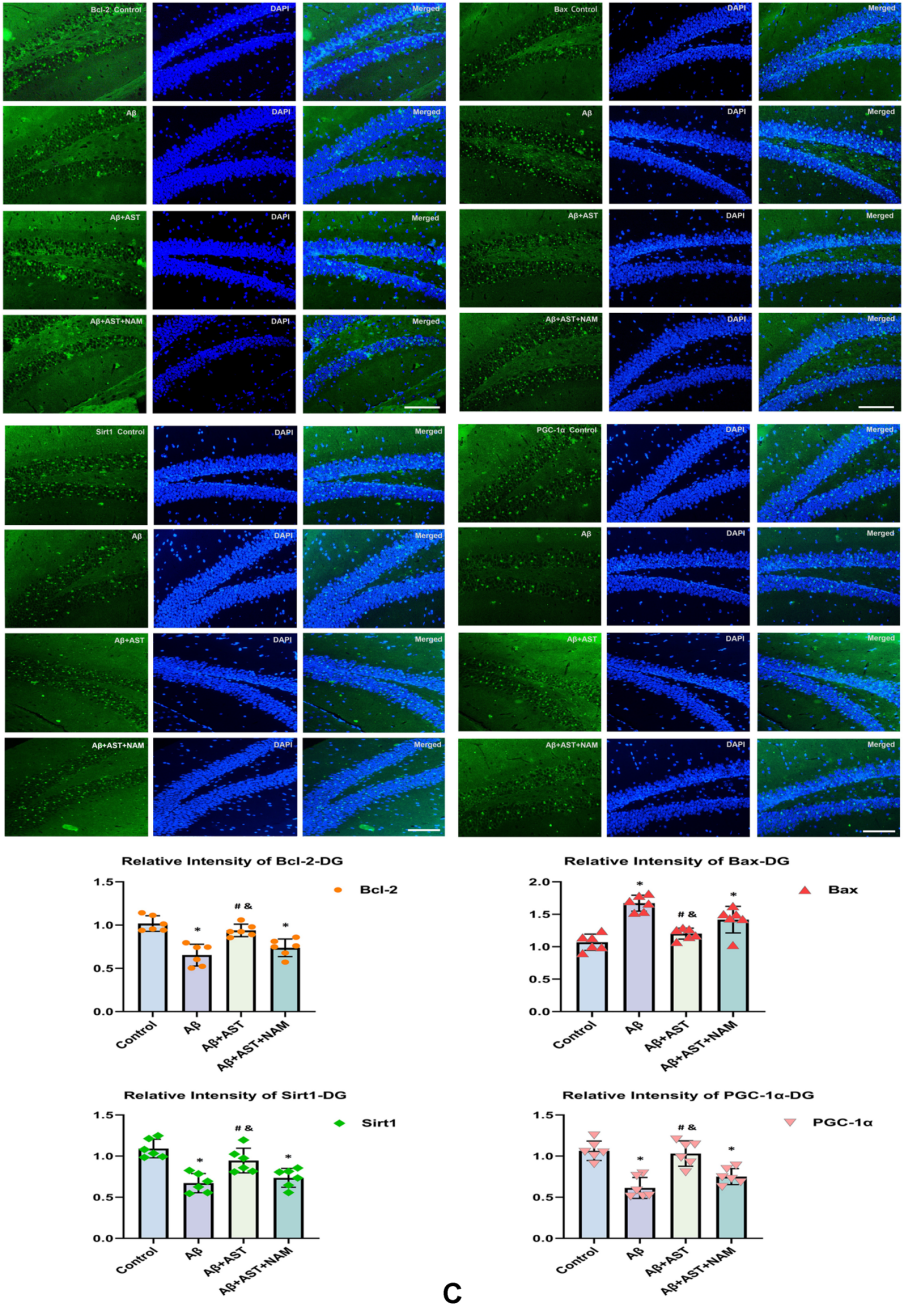
Representative images of Bcl-2, Bax, SIRT1, and PGC-1α immunofluorescence staining of three hippocampal subregions (CA1, CA3, and DG) (magnifification × 400; scale bars = 100 μm) and their quantitative analysis in **A**–**C**. *P < 0.05, Control group vs. Aβ group and Aβ + AST + NAM group. #P < 0.05, Aβ + AST group vs. Aβ + AST + NAM group. &*P* < 0.05, Aβ + AST group vs. Aβ group. (n = 6/group)

According to the immunofluorescence results, the expression levels of Bcl-2, SIRT1, and PGC-1α in the control group were higher than those in the Aβ and Aβ + AST + NAM groups. However, Bax expression was lower in the Aβ + AST group than in the Aβ and Aβ + AST + NAM groups. Notably, the expression patterns of these proteins in the hippocampus of mice were primarily observed in the DG, CA1, and CA3 subregions, and their predominant expression patterns were generally consistent across these three subregions (Fig. 3A–C).

**Fig. 4:**
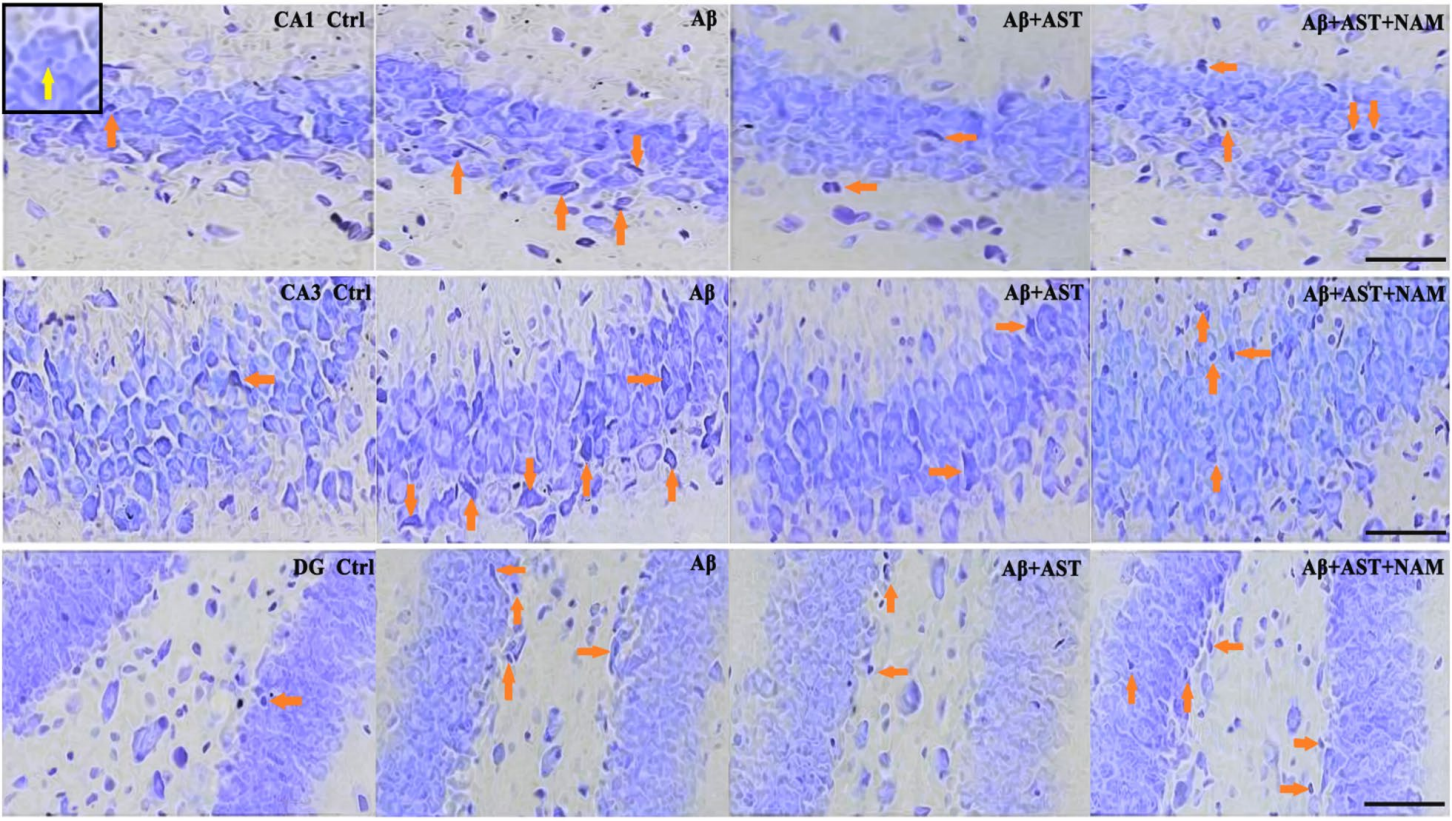
Nissl staining of hippocampal three subregions (CA1, CA3, and DG) (magnifification × 400; scale bars = 100 μm). Stained Nissl bodies can be observed in normal hippocampus neurons (yellow arrow). The degree and number of morphological changes in hippocampus neurons in each group showed significant differences (orange arrow). The neurons in the Aβ group had obvious necrosis, mainly in the form of atrophy and irregularity of neurons. The nuclei showed dark deposits. Dehydration and vacuolar structure formation occurred in the cytoplasm. The neurons in the Aβ + AST + NAM group exhibited morphological changes similar to those in the Aβ group. In the Aβ + AST group, the morphological changes in the neurons are better than that in the Aβ and Aβ + AST + NAM groups (n = 4/group)

Nissl staining was performed to observe the morphological structures of the neurons. The results showed that compared with the control group, the neurons in the Aβ group exhibited evident necrosis, with a higher number of damaged cells, primarily characterized by neuronal atrophy, irregularity, and darkly stained nuclei in addition to dehydration and vacuolar structure formation in the cytoplasm. Moreover, the hippocampi of the Aβ group showed deep staining, increased intercellular space, and nuclear atrophy (Fig. 4). Conversely, the Aβ + AST group exhibited improved neuronal body structure with reduced deep staining and atrophy, as well as less cytoplasmic dehydration and vacuole formation compared with the Aβ group. However, the structure, morphology, and staining of neurons in the Aβ + AST + NAM group showed similar abnormal changes to those observed in the Aβ group, in comparison with the control and Aβ + AST groups.

These results suggest that the reduced learning and memory ability in mice might be attributed to increased Aβ25-35-mediated neural damage, whereas AST treatment appears to alleviate the damage, leading to an improvement in learning and memory abilities.

### AST inhibited oxidative stress in the hippocampus of AD model

ROS alters and destroys intracellular molecules and increases cell membrane permeability. Therefore, oxidative stress can promote cell apoptosis by causing oxidative damage to nerve cells. SOD is a major free radical scavenger, and the higher the activity, the stronger the free radical scavenging ability. MDA content reflects the severity of cell damage and can be used as an indicator of the levels of free radicals and oxidative stress. GSH-Px is an oxygen-free radical scavenging enzyme and a major antioxidant defense component that protects cells from an increase in ROS, thereby alleviating the lipid peroxidation of polyunsaturated fatty acids in cell membranes.

**Fig. 5:**
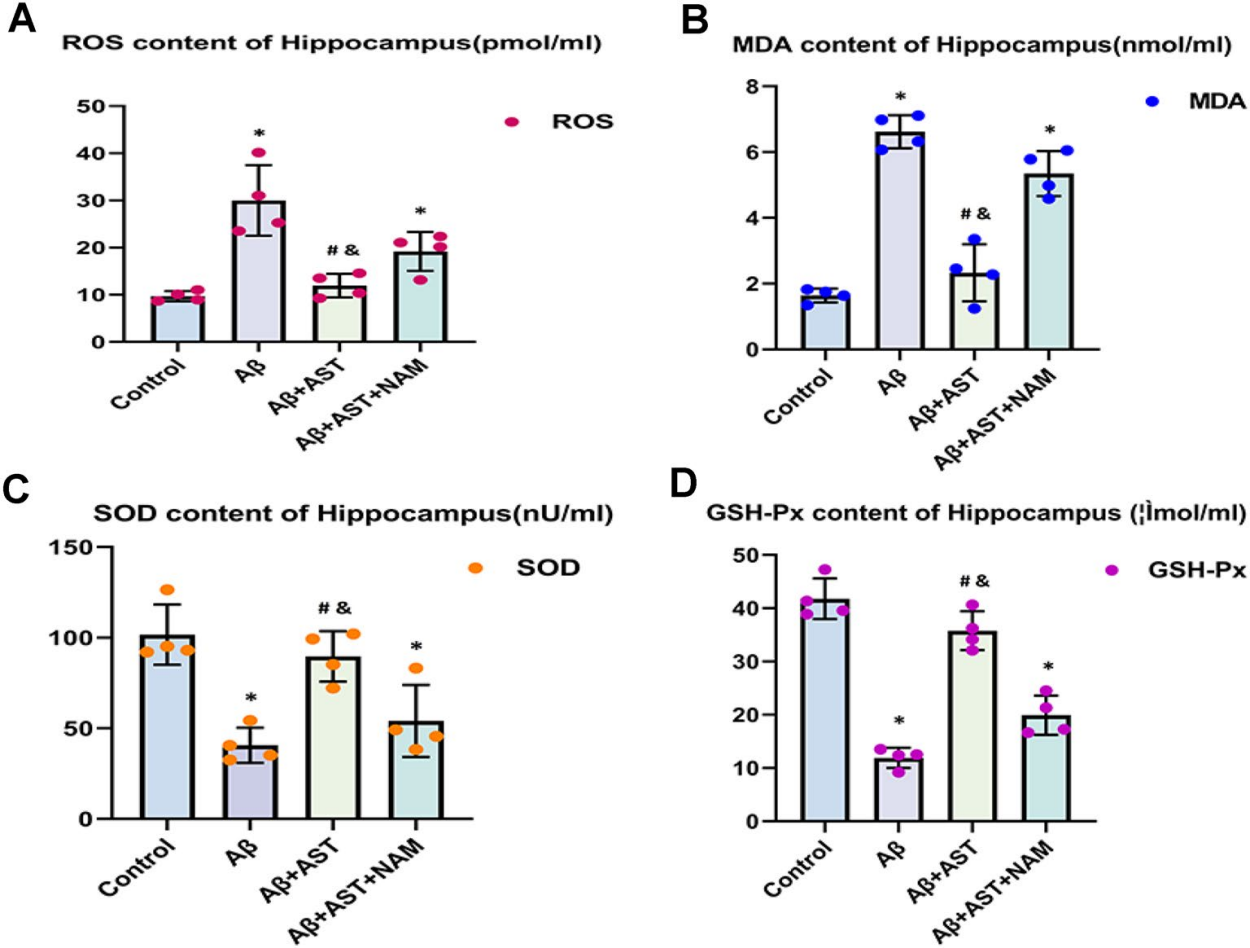
**A**–**D** Comparison of ROS, MDA, SOD, and GSH-Px results in the hippocampus of four groups of mice (n = 4/group); *P < 0.05, Control group vs. Aβ group and Aβ + AST + NAM group. #P < 0.05, Aβ + AST group vs. Aβ + AST + NAM group. &*P* < 0.05, Aβ + AST group vs. Aβ group

The expression of SOD, GSH-Px, ROS, and MDA in the hippocampus of the mice was determined using relevant test kits. The results revealed that the expression levels of ROS and MDA in the Aβ and Aβ + AST + NAM groups were greater than those in the Aβ + AST and control groups. Additionally, the expression levels of SOD and GSH-Px in the Aβ and Aβ + AST + NAM groups were lower than those in the Aβ + AST and control groups. Based on these results, activation of the SIRT1/PGC-1α signaling pathway by AST may reduce oxidative stress in the hippocampus of mice (Table 1 and Fig. 5A–D).

**Table 1:** Oxidative stress indexes of hippocampus in AD model (n = 4)

Groups	ROS	MDA	SOD	GSH-px
	(pmol/ml)	(nmol/ml)	(nU/ml)	(μmol/ml)
Control	10.05 ± 2.87	1.58 ± 0.50	95.11 ± 9.19	41.94 ± 8.71
Aβ	29.64 ± 7.07	6.58 ± 0.62	43.81 ± 12.33	14.29 ± 6.33
Aβ + AST	13.71 ± 4.12	2.83 ± 0.89	83.75 ± 14.10	34.74 ± 5.91
Aβ + AST + NAM	22.91 ± 5.71	5.27 ± 0.53	54.69 ± 14.48	18.96 ± 5.33

### AST inhibited oxidative stress in PC12 cells

**Fig. 6:**
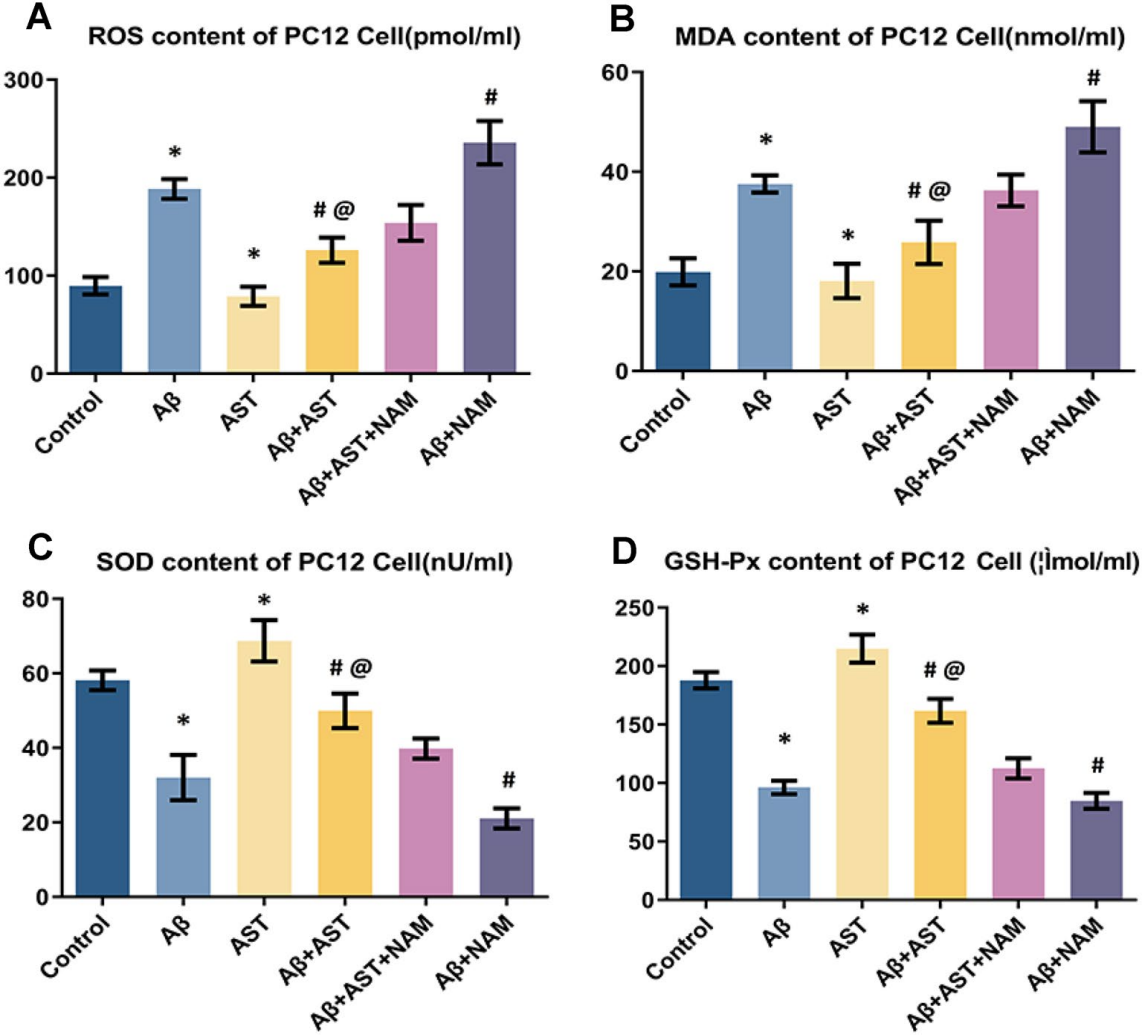
**A**–**D** Comparison of ROS, MDA, SOD, and GSH-px results of six groups of PC12 cells; *P < 0.05, Control group vs. Aβ group and AST group. #P < 0.05, Aβ + AST + NAM group vs. Aβ + AST group and Aβ + NAM group. @*P* < 0.05, Aβ + AST group vs. Aβ group

According to the results of the oxidative stress index analyses performed on PC12 cells, ROS and MDA expression levels in the Aβ + AST group were lower than those in the Aβ + AST + NAM and Aβ groups, and SOD and GSH-px expression levels were higher in the Aβ + AST group than in the Aβ + AST + NAM and Aβ groups. Specifically, SOD and GSH-px were expressed at a higher level in the Aβ + AST + NAM group compared with the Aβ + NAM group, whereas ROS and MDA were expressed at lower levels. Moreover, the control group exhibited lower SOD and GSH-px expression levels and higher ROS and MDA expression levels compared with the AST group. Furthermore, ROS and MDA expression levels were lower in the control group compared with the Aβ group; however, SOD and GSH-px expression levels were higher in the control group. These results indicate that AST suppresses oxidative stress-induced senescence in PC12 cells via the SIRT1/PGC-1α signaling pathway (Table 2 and Fig. 6A–D).

**Table 2:** Oxidative stress indexes in PC12 cells

Groups	ROS	MDA	SOD	GSH-px
	(pmol/ml)	(nmol/ml)	(nU/ml)	(μmol/ml)
Control	89.65 ± 8.87	19.88 ± 2.73	58.13 ± 3.10	187.89 ± 6.91
Aβ	188.62 ± 10.07	37.54 ± 2.04	32.03 ± 6.04	96.30 ± 5.73
AST	78.88 ± 9.77	18.07 ± 3.47	68.72 ± 5.54	217.73 ± 10.14
Aβ + AST	126.05 ± 12.82	26.25 ± 4.33	49.94 ± 5.44	161.98 ± 7.99
Aβ + AST + NAM	153.84 ± 18.22	37.02 ± 3.18	39.81 ± 2.86	112.53 ± 8.61
Aβ + NAM	235.81 ± 22.14	50.36 ± 5.14	21.08 ± 3.17	84.41 ± 4.89

### AST inhibited the apoptosis of PC12 cells

#### CCK-8 assays were used to detect the activity of PC12 cells

**Fig. 7:**
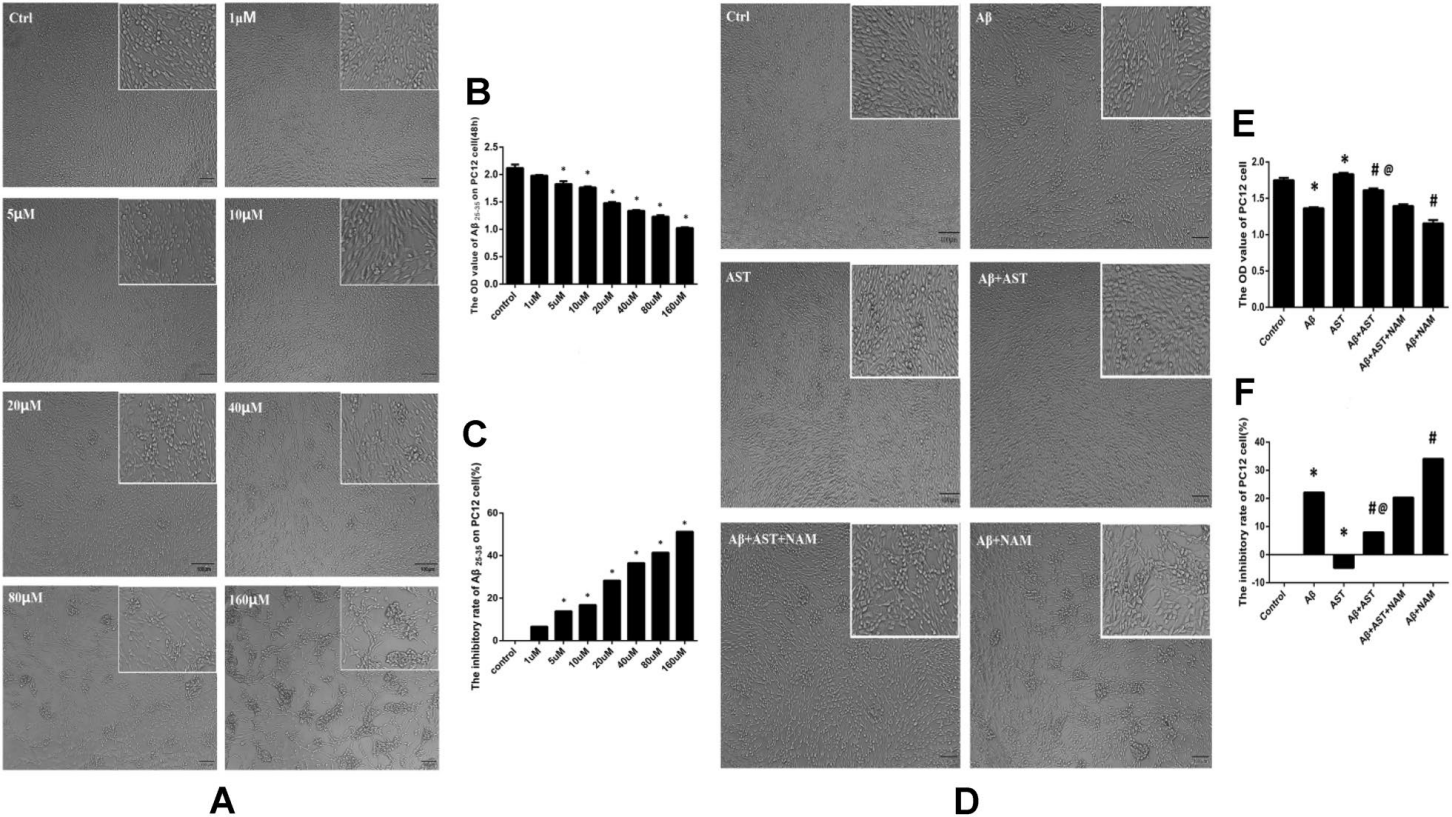
**A** Results of CCK8 assays for the toxicity of Aβ25-35 concentrations on PC12 cells (bar = 100 µm). **B**–**C** OD values of eight groups of PC12 cells, and inhibition rate of Aβ25-35 in each group on PC12 cells. *P* > 0.05, the Control group vs. 1 µm group, there was no significant difference in OD value and cell inhibition rate. *P < 0.05, the Control group vs. other groups. **D** Results of CCK8 assay for the toxicity of Aβ25-35 concentrations on PC12 cells (bar = 100 µm). **E**–**F** OD values of six groups of PC12 cells, and inhibition rate of Aβ25-35 in each group on PC12 cells.*P < 0.05, Control group vs. Aβ group and AST group; #*P* < 0.05, Aβ + AST + NAM group vs. Aβ + AST group and Aβ + NAM group; @*P* < 0.05, Aβ + AST group vs. Aβ group

CCK-8 assays revealed that at a dose concentration of 1 μM, cells treated with Aβ25–35 did not exhibit a statistically significant difference compared with the control group cells (*P* > 0.05), indicating that this concentration was not cytotoxic to PC12 cells. However, cells treated with Aβ25–35 at concentrations of 5 μM, 10 μM, 20 μM, 40 μM, 80 μM, and 160 μM demonstrated statistically significant differences compared with the control group (*P* < 0.05). The findings indicate that Aβ25–35 is hazardous to cells when administered for 48-h at a concentration of 5 μM. Additionally, the inhibition rate of PC12 cells increased significantly with higher concentrations of Aβ25–35. In this investigation, the maximum tolerated dose concentration of Aβ25–35 was 20 μM, as concentrations of 40, 80, and 160 μM resulted in cell inhibition rates close to or exceeding 50% (Fig. 7A–C).

In the control group, cells exhibited favorable characteristics, adhering to the wall, exhibiting uniform expansion, and assuming polygonal shapes, as indicated by the outcomes of the CCK-8 assay. However, the Aβ group displayed poor cell conditions, with uneven density and irregular shapes. Conversely, the AST group showed higher cell density and more regular morphology than the control group. The Aβ + AST group exhibited a denser cell population and more regular cell shapes compared with the Aβ + AST + NAM group. Additionally, the Aβ + NAM group cells appeared erratic and were significantly fewer in number.

Moreover, the CCK-8 results revealed that the Aβ group cell viability was lower than that of the control group. In the Aβ + AST group, Aβ had no significant effect on cell viability, which remained at the same level as that of the control group. However, the AST group showed improved cell viability. These findings suggest that AST may provide protection against Aβ-mediated cytotoxicity. Moreover, the cell activity of the Aβ + AST group was higher than that of the Aβ + AST + NAM group, whereas that of the Aβ + AST + NAM group was significantly lower, indicating that AST can prevent the cytotoxicity induced by Aβ25–35-mediated oxidative stress through the SIRT1/PGC-1α signaling pathway, thereby exerting a protective effect on PC12 cells (Fig. 7D–F).

#### Annexin V/PI double staining was used to detect the apoptosis of PC12 cells

**Fig. 8:**
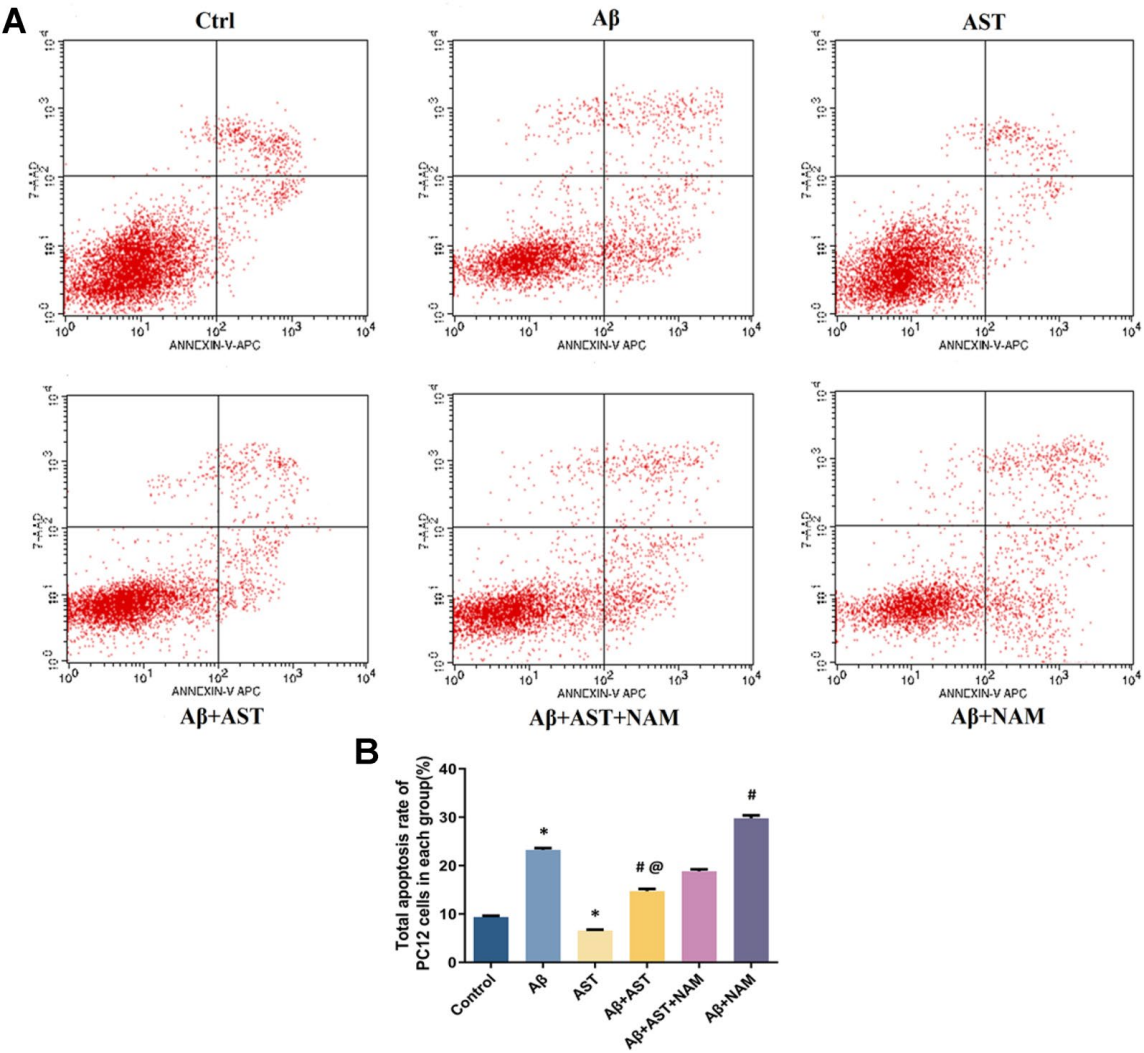
**A** Apoptosis status of six groups of PC12 cells in each quadrant. **B** Comparison of total apoptosis rate of PC12 cells in six groups. *P < 0.05, Control group vs. Aβ group and AST group; #*P* < 0.05, Aβ + AST + NAM group vs. Aβ + AST group and Aβ + NAM group; @*P* < 0.05, Aβ + AST group vs. Aβ group

Our findings showed that the apoptosis rate in the control group was 9.35%. In contrast, the apoptosis rate of the Aβ group was 23.25%, indicating a substantial upward trend. However, the apoptosis rate in the AST group was notably lower at 6.59% compared with the control group. Additionally, the apoptosis rate of the Aβ + AST group was 10.35%, which was statistically different from the Aβ group, and the apoptosis rate of the Aβ + NAM group was 29.80%, which was also statistically different from the Aβ + AST + NAM group. The findings demonstrate that Aβ25–35 significantly increases the likehood of apoptosis in PC12 cells, while AST administration mitigates this process by reducing the rate of apoptosis in PC12 cells. Furthermore, AST demonstrates the ability to protect against cytotoxic damage induced by Aβ25–35, preventing PC12 cells from undergoing apoptosis through the SIRT1/PGC-1α signaling pathway (Table 3 and Fig. 8A–B).

**Table 3:** Apoptotic rate of PC12 cells

Cell states (%)	Apoptotic rate (%)					
	Control	Aβ	AST	Aβ + AST	Aβ + AST + NAM	Aβ + NAM
UL (%)	0.81	2.61	0.69	2.51	2.43	1.58
UR (%)	5.60	17.87	3.94	4.90	6.94	11.44
LL (%)	89.84	74.21	92.72	87.14	78.75	68.62
LR (%)	3.75	15.38	2.66	5.45	11.87	18.36
Total apoptosis rate	9.35	23.25	6.59	10.35	18.82	29.80

#### β -galactosidase staining was used to analyze the senescence of PC12 cells

**Fig. 9:**
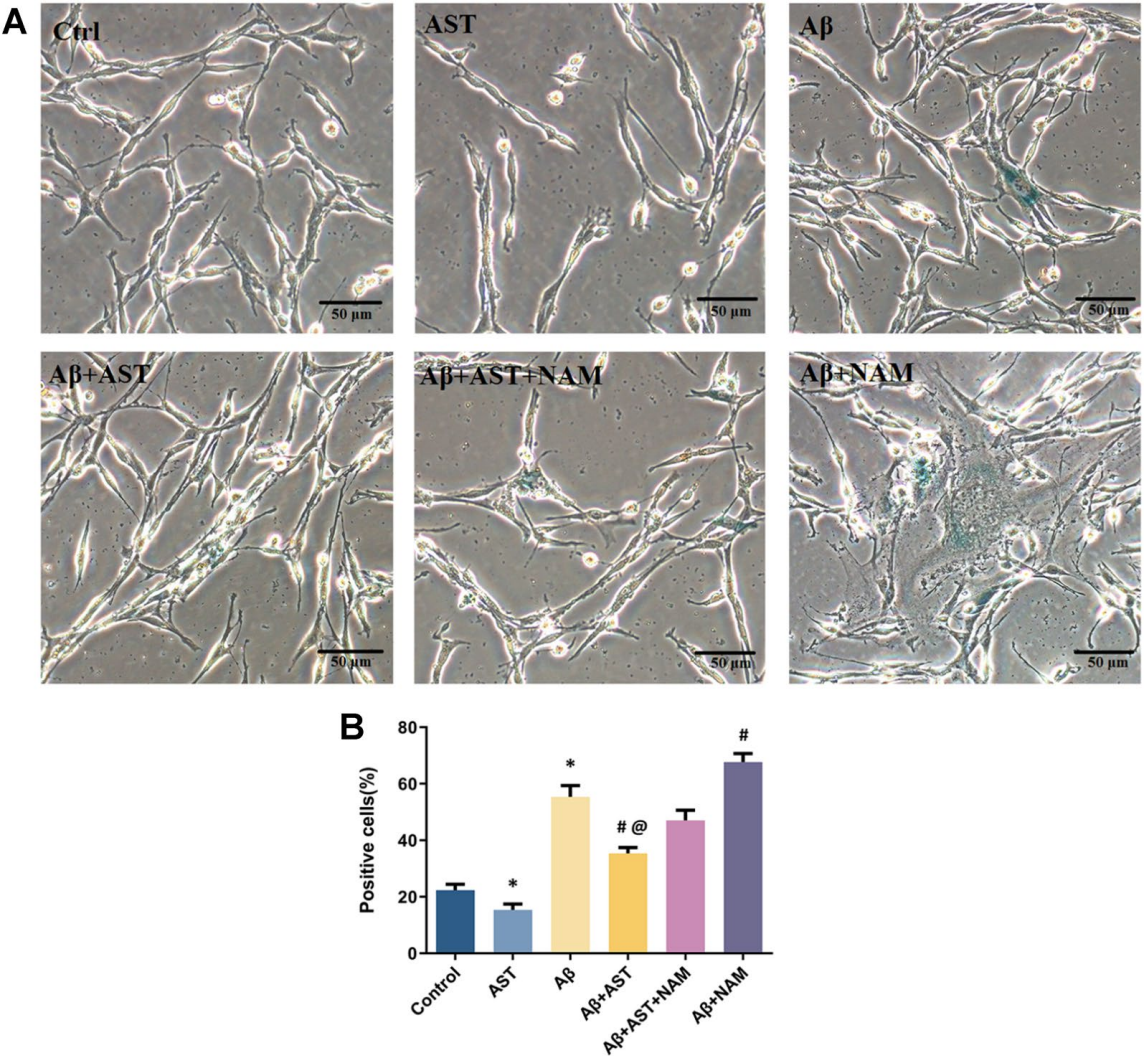
**A** Results of β-galactosidase staining of six groups of PC12 cells (bar = 50 µm). Comparison of β-galactosidase staining results. *P < 0.05, Control group vs. Aβ group and AST group; #*P* < 0.05, Aβ + AST + NAM group vs. Aβ + AST group and Aβ + NAM group; @*P* < 0.05, Aβ + AST group vs. Aβ group

The average percentage of β-Gal-positive cells in the control group was 22%, whereas that in the Aβ group was 55%. Compared with the control group, 15% of the cells in the AST group were positively stained. Additionally, the Aβ + AST group had 35% more positively stained cells, whereas the Aβ + NAM group had 78%, compared with the Aβ and Aβ + AST + NAM groups. These findings demonstrate that Aβ could induce senescence in PC12 cells and influence cell activity. Nevertheless, AST prevented the senescence of PC12 cells via the SIRT1/PGC-1α signaling pathway and acted as a protective agent against cytotoxic damage caused by Aβ25–35(Fig. 9A–B).

## Discussion

### Effects of AST on senescence and apoptosis of hippocampus neurons in AD model

With aging, the brains becomes more vulnerable to early onset of neurodegenerative and neuroinflammation [35, 36]. This gradual aging process of the brain give rise to significant changes, particularly in brain regions linked to learning and memory, resulting in a decline in cognitive abilities and memory functions. Consequently, neurodegenerative conditions such as AD manifest as the brain ages.

The accumulation of Aβ, the primary component of amyloid plaques, in and around the cerebrovascular system is a pathogenic characteristic of AD and may lead to damage to the blood–brain barrier [37, 38]. Conversely, AST is a potent antioxidant that reduces inflammation and DNA damage while preventing cell damage caused by elevated ROS [39]. The capacity of AST to halt oxidative damage in the central nervous system is owing to its distinctive molecular structure, which quenches singlet oxygen and scavenges free radicals [40]. In the present study, the results of our mouse model experiment demonstrate that Aβ can decrease hippocampal neuronal density and trigger apoptosis in mice, thereby decreasing their capacity for spatial learning and memory. Furthermore, we found that Bcl-2, SIRT1, and PGC-1α expression was increased by AST, whereas Bax expression was decreased, demonstrating that AST also possesses antioxidant properties in addition to controlling oxidative stress indicators.

The SIRT1/PGC-1α signaling pathway is closely related to changes in cellular antioxidants, apoptosis, and mitochondrial function [41–43]. In our in vivo experiments, hematoxylin–eosin staining was applied to the CA1, CA3, and DG subregions of the mice hippocampus to observe the pyramidal cell morphological changes in the hippocampus. However, in addition to the previously investigated Bcl-2 and Bax, further studies into changes in the expression of downstream protein caspase 3, a component of apoptosis-related proteins, are required to verify the inhibitory effect of AST on cell apoptosis. This approach parallels analogous studies focused on cell apoptosis [44].

**Fig. 10:**
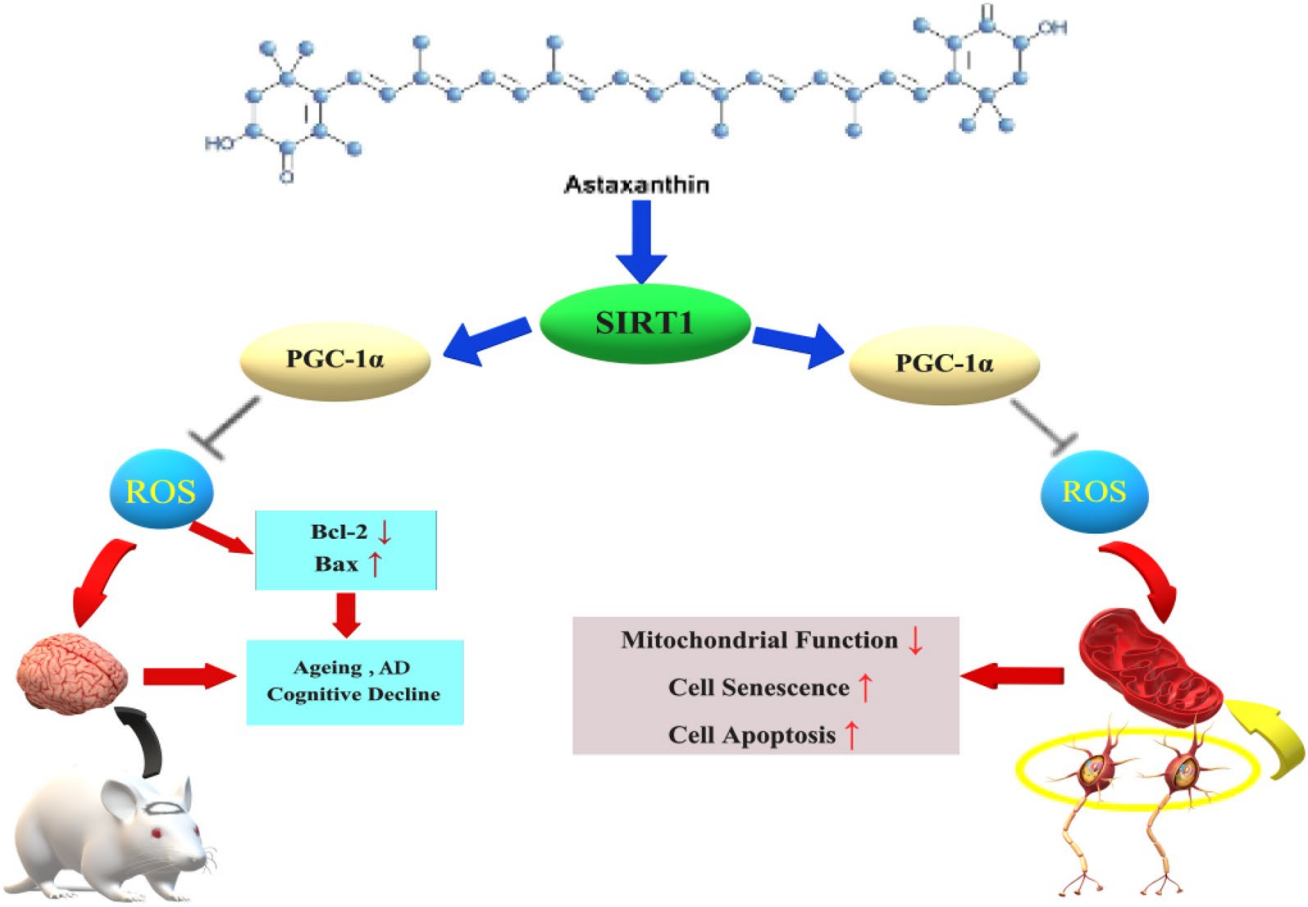
The diagram illustrates the role of astaxanthin in the development of senescence and apoptosis of neurons. ROS production increased after Aβ25-35 treatment. In vivo experiments, ROS can reduce the expression of Bcl-2 and increase the expression of Bax in the hippocampus of mice, which can promote the senescence and apoptosis of neurons, accelerate the process of brain aging and AD, and reduce cognitive function. In vitro experiments, ROS reduced mitochondrial function, senescence, and apoptosis of PC12 cells. Astaxanthin and SIRT1, PGC-1α have strong antioxidant effects, which can resist cell senescence and apoptosis caused by oxidative stress, improve mitochondrial function, and improve the cognitive function of mice

NAM can inhibit SIRT1 and other members of the sirtuin family, including SIRT2, SIRT3, and SIRT6. NAM is a precursor of NAD + and influences the activity of various enzymes that utilize NAD + as a coenzyme. However, this study focused only on the inhibitory effect of NAM on SIRT1 expression. Therefore, in future studies, we plan to explore whether NAM exerts inhibitory effects on other members of the sirtuin protein family. Although our experimental results indicate that NAM plays a role in inhibiting SIRT1, some studies have suggested that NAM can promote the expression of SIRT1 [45, 46]. Therefore, the mechanism underlying the effect of NAM on SIRT1 remains unclear. However, the results obtained from our in vivo experiments suggest that AST could decrease the neuronal mortality caused by oxidative stress generated by Aβ25–35 through its ability to enhance the activity of the SIRT1/PGC-1α signaling pathway. Additionally, AST has a significant antioxidant effect and acts as a protective agent against neurodegenerative diseases, ultimately leading to an enhancement in the cognitive function of mice (Fig. 10).

### Protective effects of AST on Aβ25–35-induced senescence and apoptosis of PC12 cells

Aging is a complex physiological process characterized by the steady decline in cellular function and an increased likelihood for cell death. Oxidative stress plays a pivotal role in brain aging, leading to senescence and apoptosis in various mammalian cell lines when exposed to high levels of oxidative stress [47]. To counteract this, cells rely on antioxidant enzymes, with carotenoids being one of the primary cellular defense mechanisms. Carotenoids are a physically and functionally diverse collection of natural pigments that are potent antioxidants capable of removing monomorphic oxygen and peroxyl radicals from humans and other animals [48]. Among these carotenoids, AST, derived from seaweed, has garnered particular attention as a powerful antioxidant [49]. In some studies, following treatment with AST, cell growth resumed, and chromatin concentration and nuclear fragmentation were reduced, demonstrating that the anti-apoptotic, anti-neuroinflammatory, and anti-aging properties of AST can protect cells against oxidative stress-induced apoptosis [50–52].

The results of the CCK-8 assay, double Annexin-V/PI staining, β-Gal staining, and oxidative stress index analysis indicated that Aβ substantially promoted the senescence and apoptosis of PC12 cells. However, AST treatment inhibited the induced senescence and apoptosis of PC12 cells. Moreover, the resistance of PC12 cells to ROS increased following AST treatment, indicating enhanced cellular activity. Our findings indicate that the SIRT1/PGC-1α signal pathway plays a significant role in enhancing anti-oxidation and anti-aging activities within cells. Moreover, after the addition of NAM, an inhibitor of the SIRT1/PGC-1α signaling pathway, the activity of PC12 cells decreased, the apoptosis rate increased, the number of β-Gal-positive cells increased, the levels of ROS and MDA increased, and the levels of SOD and GSH-Px decreased. Furthermore, as cellular senescence and apoptosis are closely related to decreased mitochondrial function, it is imperative for future research to also center on alterations in mitochondrial membrane potential and mitochondrial permeability transition pores.

According to our findings, AST can inhibit the production of ROS via the SIRT1/PGC-1α signaling pathway, thereby enhancing its antioxidant effect, preventing oxidative stress-induced cell senescence and apoptosis, and reducing the risk of age-related diseases. Using the PC12 cell model, this study has demonstrated the promising potential of AST as a neuroprotective agent against neurodegenerative diseases. Moreover, it provides a crucial theoretical foundation and serves as a valuable therapeutic target for further investigations into the antioxidant and anti-brain aging properties of AST (Fig. 10).

## Conclusions

AST effectively regulates and inhibits Aβ25–35-mediated oxidative stress–induced apoptosis of hippocampal neurons in AD mice through the SIRT1/PGC-1α signaling pathway. AST plays a protective role in neurons and enhances learning, memory, and cognitive abilities. Additionally, AST curtails senescence and apoptosis in PC12 cells. Further research is required to investigate the antioxidant mechanism of AST and explore its additional beneficial effects in both in vivo and in vitro settings.

## Data Availability

The data that support the findings of this study are available from the corresponding author upon reasonable request.

## Abbreviations

Aβ: β-Amyloid
AD: Alzheimer’s disease
AST: Astaxanthin
Bax: Bcl2-Associated X
Bcl-2: B-cell lymphoma-2
β-Gal: β-Galactosidase
CCK8: Cell Counting Kit-8
CNS: Central nervous system
GSH-px: Glutathione peroxidase
MDA: Malondialdehyde
MWM: Miorrs water maze
NAM: Nicotinamide
PC12: Pheochromocytomaderived cell
PGC-1α: Peroxisome proliferator-activated receptor γ coactiva-tor-1
ROS: Reactive Oxygen Species
SIRT1: Silent Information Regulator 1
SOD: Superoxide dismutase
